# Malaria surveillance from both ends: concurrent detection of *Plasmodium falciparum* in saliva and excreta harvested from *Anopheles* mosquitoes

**DOI:** 10.1186/s13071-019-3610-9

**Published:** 2019-07-18

**Authors:** Ana L. Ramírez, Andrew F. van den Hurk, Ian M. Mackay, Annie S. P. Yang, Glen R. Hewitson, Jamie L. McMahon, Justin A. Boddey, Scott A. Ritchie, Sara M. Erickson

**Affiliations:** 1 College of Public Health, Medical and Veterinary Sciences, James Cook University, PO Box 6811, Cairns, QLD 4870 Australia; 2 Australian Institute of Tropical Health and Medicine, James Cook University, PO Box 6811, Cairns, QLD 4870 Australia; 3 Public Health Virology, Forensic and Scientific Services, Department of Health, Queensland Government, Coopers Plains, QLD 4108 Australia; 4 Infection and Immunity Division, The Walter and Eliza Hall Institute of Medical Research, Parkville, VIC 3052 Australia; 5 Department of Medical Biology, The University of Melbourne, Parkville, VIC 3052 Australia; 6 Present Address: Department of Medical Microbiology Parasitology, Radboud University Medical Center, Geert Grooteplein 28, Microbiology 268, 6500 HB Nijmegen, The Netherlands

**Keywords:** Malaria, Mosquito, Saliva, Excreta, Sporozoite, *Plasmodium falciparum*, *Anopheles stephensi*

## Abstract

**Background:**

Malaria is the most important vector-borne disease in the world. Epidemiological and ecological studies of malaria traditionally utilize detection of *Plasmodium* sporozoites in whole mosquitoes or salivary glands by microscopy or serological or molecular assays. However, these methods are labor-intensive, and can over- or underestimate mosquito transmission potential. To overcome these limitations, alternative sample types have been evaluated for the study of malaria. It was recently shown that *Plasmodium* could be detected in saliva expectorated on honey-soaked cards by *Anopheles stephensi*, providing a better estimate of transmission risk. We evaluated whether excretion of *Plasmodium falciparum* nucleic acid by *An. stephensi* correlates with expectoration of parasites in saliva, thus providing an additional sample type for estimating transmission potential. Mosquitoes were exposed to infectious blood meals containing cultured gametocytes, and excreta collected at different time points post-exposure. Saliva was collected on honey-soaked filter paper cards, and salivary glands were dissected and examined microscopically for sporozoites. Excreta and saliva samples were tested by real time polymerase chain reaction (RT-rtPCR).

**Results:**

*Plasmodium falciparum* RNA was detected in mosquito excreta as early as four days after ingesting a bloodmeal containing gametocytes. Once sporogony (the development of sporozoites) occurred, *P. falciparum* RNA was detected concurrently in both excreta and saliva samples. In the majority of cases, no difference was observed between the C_t_ values obtained from matched excreta and saliva samples, suggesting that both samples provide equally sensitive results. A positive association was observed between the molecular detection of the parasites in both samples and the proportion of mosquitoes with sporozoites in their salivary glands from each container. No distinguishable parasites were observed when excreta samples were stained and microscopically analyzed.

**Conclusions:**

Mosquito saliva and excreta are easily collected and are promising for surveillance of malaria-causing parasites, especially in low transmission settings or in places where arboviruses co-circulate.

**Electronic supplementary material:**

The online version of this article (10.1186/s13071-019-3610-9) contains supplementary material, which is available to authorized users.

## Background

Malaria is the deadliest vector-borne disease, with an estimated 219 million cases and 435,000 deaths in 2017 alone [1]. More than 90% of the cases occur in sub-Saharan Africa, and children under five years are the most vulnerable group. *Plasmodium falciparum* is the most prevalent causative agent of human malaria and has the most severe clinical manifestations [2]. The parasites are transmitted to humans by anopheline mosquitoes. More than 70 *Anopheles* species are competent vectors of malaria and more than half of these are responsible for transmitting the majority of human malaria parasites [3, 4].

Malaria control, elimination and ultimately, eradication, are global priorities, with 21 countries committed to eliminate malaria by 2020 [5]. Malaria elimination is achieved through a combination of antimalarial treatments (such as artemisinin-based combination therapy, ACT), vector control and source reduction of mosquito larval habitats. Surveillance is a crucial component of malaria intervention programmes, providing information to guide initiatives and measure their impact [6] and is regarded as one of the three fundamental pillars of the Global Technical Strategy [7]. Malaria surveillance strategies are dependent on the level of transmission, where lower levels of transmission require increased efforts to detect new cases and transmission foci. Generally, malaria surveillance focuses on passive or active case detection, monitoring of anti-malarial drug resistance and entomological surveillance, including detection of insecticide resistance [8].

Detection of *Plasmodium* in mosquitoes is an essential parameter used to estimate metrics of exposure and transmission intensity. The sporozoite, the infectious stage of the parasite in the mosquito is usually the target of these efforts. There are several approaches to detect sporozoites in field-collected mosquitoes. Traditionally, their salivary glands are dissected and observed under a compound microscope for the presence of sporozoites [9, 10]. Alternatively, enzyme-linked immunosorbent assays (ELISAs) have been used to detect sporozoite protein in salivary glands or pools of mosquitoes [11, 12]. Rapid diagnostic tests in dipstick format have also been developed [13, 14], with results comparable to those obtained by ELISA [15]. A variety of polymerase chain reaction (PCR) methods are available, with high sensitivity and versatility [16–18]. However, although certainly useful, these techniques have limitations. Dissection and observation of salivary glands is time-consuming, require skill and expertise, can fail to detect infections with low numbers of sporozoites and is not species specific. Immunoassays can yield false positives that need to be confirmed by molecular methods [10, 19, 20]. Even though PCR assays allow for high-throughput analysis, the identification and sorting of mosquitoes can be labor-intensive, especially for larger collections. They also require specialized facilities, equipment and expertise, which is often not available in low resource settings. Finally, all these methods can overestimate transmission, since not all the sporozoites present in the salivary glands will be ejected by a feeding mosquito [21].

Analysis of mosquito saliva for the presence of pathogens provides a better estimate of transmission risk. Mosquito saliva has been used for the study of other mosquito-borne diseases, particularly arthropod-borne viruses (arboviruses), both in the field and the laboratory [22, 23]. It had been demonstrated that *P. falciparum* could be detected in mosquito saliva collected by forced salivation [9], but it was not until recently that *P. falciparum* sporozoites were detected in saliva expectorated on honey-soaked nucleic acid preservation cards, allowing for detection without killing the mosquito [24]. Alternatively, mosquito excreta has emerged as a promising sample for the study of arboviruses [25–27], filarial parasites and malaria [28]. Mosquito excreta has the added potential to be used for xenomonitoring, where the mosquitoes are used as “flying syringes” to sample vertebrate hosts to monitor human and animal diseases and methodologies are being developed to collect mosquito excreta in the field [29].

The primary objective of the current study was to determine, through proof of concept, if *P. falciparum* could be detected by molecular assays concurrently in excreta and saliva of *Anopheles stephensi* mosquitoes. We also correlated the detection of the parasite in excreta and saliva with salivary gland sporozoite infection in the mosquitoes. Finally, we analyzed excreta samples microscopically for evidence of recognizable parasites.

## Methods

### Parasite maintenance

The asexual stages of *P. falciparum* NF54 were maintained at 4% hematocrit in human O-positive erythrocytes (Australian Red Cross, Melbourne) in RPMI-HEPES with 10% heat-inactivated human serum (Australian Red Cross, Melbourne) in an atmosphere of 94% N, 5% CO_2_, 1% O_2_ [30]. Gametocytes were generated as described previously, using the crash method [31]. After 17 days, gametocytes were quantified by Giemsa smears, harvested, and five different blood meals prepared by dilution to 0.3% stage V gametocytemia in human serum for feeding to mosquitoes [30].

### Mosquito rearing

Experiments were performed using *Anopheles stephensi* mosquitoes (John Hopkins School of Public Health strain) at the Walter and Eliza Hall Institute of Medical Research. Larvae were fed a 1:1 ratio of TetraMin^®^ and Nutrafin^®^ Max tropical fish food flakes. After adult emergence, mosquitoes were provided sugar cubes and water in a cotton wick *ad libitum*. Females were offered mouse blood in water-jacketed, glass membrane feeders (Lillie Glassblowers, Inc., Georgia, USA) to stimulate egg production. All mosquitoes were maintained at 26 °C, 80% RH and 12:12 L:D for the duration of the study.

### Exposure of mosquitoes to *P. falciparum* gametocyte cultures and analysis of parasite development

Four- to five-day-old mosquitoes were deprived of sugar overnight (10–14 h) prior to being exposed to *P. falciparum* gametocytes. Females were aspirated into 0.946 l paper cartons (Castaway Food Packaging, Australia) secured with mesh lids where they were offered a gametocytemic blood meal through a water-jacketed, glass membrane feeder. Two hours after feeding, mosquitoes were CO_2_ anesthetized and sorted on wet ice. Only fully engorged females were maintained, whilst males, non-fed and partially-fed females were discarded. Fully engorged females were immediately placed in a 24.5 cm^3^ mesh cage (Bugdorm-42222, Bugdorm, Taichung, Taiwan), with sugar cubes and a water wick, or in modified containers for excreta collection (see below). At day 8 post-exposure (PE), the midguts from 16–23 cold-anesthetized and ethanol-killed mosquitoes from each cohort were dissected and stained with 0.1% mercurochrome (w/v) in water, and oocysts per mosquito enumerated by microscopy. At day 17 PE, 30–32 salivary glands from mosquitoes from each cohort were dissected and pooled before being homogenized in PBS with a pestle to release sporozoites. After filtering through glass wool, sporozoites were counted using a Neubauer hemocytometer, and each cohort sample was counted in triplicate.

### Collection of mosquito excreta and saliva

Two experiments were conducted to evaluate the use of mosquito excreta and saliva for *P. falciparum* detection. In the first experiment, groups of mosquitoes were followed over time to establish the time of first detection in excreta. For this, 20 batches of 5 mosquitoes which had been exposed to two different gametocytemic blood meals were placed in modified 150 ml polypropylene containers for excreta collection [26]. The containers had a fiberglass insect screen floor to allow excreta to pass through onto a parafilm disc and the top opening of the containers was covered in mesh. Mosquitoes were maintained on cotton pledgets soaked in 15% honey water dyed with blue food coloring for excreta visualization. Excreta was collected daily from day 4 to 14 PE using a cotton swab moistened with PBS. Swabs were placed in a 1.5 ml tube with 500 µl PBS and stored at − 80 °C. Cotton pledgets and parafilm discs were replaced daily to avoid cross-contamination and mortality was recorded daily.

In the second experiment, from day 15 to 19 PE, excreta and saliva were collected from groups of mosquitoes and the presence of sporozoites in their salivary glands was visually assessed. For this, 3 groups of 5 mosquitoes from cohorts that had fed on 5 different blood meals containing gametocytes were placed in modified containers as described above. For daily saliva collection, mosquitoes were allowed to feed on a 4 cm^2^ filter paper card (FP; low chamber filter paper, Bio-Rad Laboratories, California) soaked in 100% honey dyed with blue food coloring. After 24 h, excreta was collected as previously described, whilst the FP cards were removed and placed in a 1.5 ml tube containing 0.5 mL PBS and stored at − 80 °C. The mosquitoes were CO_2_ anesthetized, ethanol-killed, and the salivary glands dissected and assessed for the presence of sporozoites using a compound microscope. The sporozoite rate of the container was calculated as the number of mosquitoes with sporozoites in their salivary glands per the number of surviving mosquitoes in the container.

### Detection of *Plasmodium* spp. by real-time RT-rtPCR

Thawed excreta samples were agitated using a Qiagen Tissue Lyser II (Qiagen, Hilden, Germany) for 3 min at 26 Hz and centrifuged for 1 min at 14,000×*g* [26]. Thawed FP cards were maintained at 4 °C and briefly vortexed every 5 min for 20 min [22]. RNA was extracted from excreta samples and FP card eluates using a QIAmp One-For-All Nucleic Acid Kit (Qiagen, Hilden, Germany) in a QIAxtractor (Qiagen, Hilden, Germany) according to the manufacturer’s instructions. A Taqman^®^ real-time RT-PCR (RT-rtPCR) assay (modified from [32]) was used to detect *Plasmodium* spp. The assay amplifies a conserved region of the *18S* rRNA gene. The primers and probe were: forward primer (5′-AGG AAG TTT AAG GCA ACA ACA GGT-3′); reverse primer (5′-GCA ATA ATC TAT CCC CAT CAC GA-3′); and probe (5′-6FAM-TGT CCT TAG ATG AAC TAG GCT GCA CGC G-BHQ-1-3′). Primer and probe oligonucleotides were synthesized by Sigma-Aldrich (Castle Hill, Australia). The reaction mix was prepared using SuperScript III Platinum^®^ one-step quantitative RT-PCR system (Invitrogen, Carlsbad, CA) and contained 0.4 µl of SuperScript^®^ III/Platinum^®^ *Taq* mix, 10 µl of 2× reaction mix, 50 nM of ROX reference dye, primers and probe in a final optimized concentration of 900 nM and 150 nM respectively, 5 μl of extracted RNA and nuclease-free water to produce a final volume of 20 µl. The assays were run in a Rotor-Gene 6000 real-time PCR cycler (Qiagen, Australia) with cycling conditions as follows: (i) one cycle at 50 °C for 5 min; (ii) one cycle at 95 °C for 2 min; and (iii) 50 cycles of 95 °C for 3 s and 60 °C for 30 s. Each run included a positive extraction control (bovine viral diarrheal virus, BVDV) and a positive *P. falciparum* control extracted from sporozoites; a negative extraction control and a no-template control (molecular grade water). The cycle threshold number (C_t_) was determined for each sample; any sample with a C_t_ > 40 was considered negative. To determine the assay’s limit of detection, a sample of quantified sporozoites from salivary glands was extracted as described above, and 10-fold dilutions were used to generate a standard curve with undiluted RNA and each dilution (10^−1^ to 10^−8^) tested in triplicate.

### Visualization of *P. falciparum* in mosquito excreta

A total of six aliquots from 10 excreta samples that were positive by RT-rtPCR were air-dried, methanol-fixed and dyed with 11% Giemsa stain diluted in distilled water for 60 min before being washed with water, dried and examined using a compound microscope under 1000× magnification.

### Statistical analyses

All data sets were tested for normality using Shapiro-Wilks tests. Differences in salivary gland infection between cohorts were analyzed using a one-way ANOVA followed by Tukey’s multiple *post-hoc* comparison test. Differences in oocyst counts between cohorts and C_t_ values for excreta and expectorate between days and between groups were analyzed using the Kruskal-Wallis test followed by Dunn’s multiple comparison test. Differences in C_t_ values between excreta and saliva were analyzed using the Wilcoxon matched-pairs signed rank test. Differences between the proportion of positive saliva and excreta samples were analyzed using the Fisher’s exact test. Associations between sporozoite rates and C_t_ values from saliva and excreta were analyzed using Spearman’s rank correlation. All figures, Kaplan-Meier survival curves and statistical analyses were performed using GraphPad Prism version 7.0c (GraphPad Software, La Jolla, CA, http://www.graphpad.com).

## Results

### Parasite development in the mosquito

**Fig. 1:**
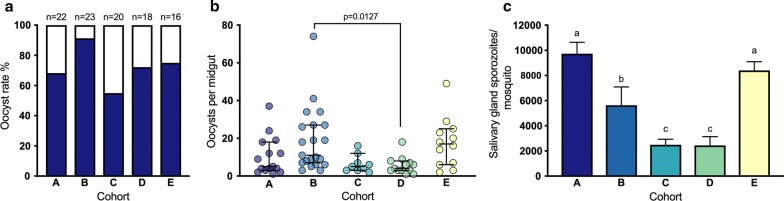
Parasite development in mosquito cohorts exposed to five different bloodmeals (indicated as cohorts **a**–**e**). **a** Proportion of mosquitoes with oocysts in their midguts at day 8 PE. **b** Oocyst counts per mosquito midgut 8 days PE (median and 95% CI, Kruskal-Wallis one-way ANOVA followed by Dunn’s multiple comparison test). Each dot corresponds to one midgut. **c** Salivary gland sporozoite loads per mosquito 17 days PE. Different letters indicate statistically significant differences between groups (mean ± SEM, *n* = 4, one-way ANOVA test followed by Tukey’s multiple comparison test, *P* < 0.05)

There was no difference in mosquito survival distributions between cohorts (Log-Rank statistic $\chi^{ 2}_{\left( 3\right)}$ = 4.415, *P* = 0.220; Additional file 1: Figure S1). The overall oocyst rate (prevalence of mosquito infection) in mosquito midguts at day 8 PE was 72.7%, ranging from 55% for cohort C to 91% for cohort B (Fig. 1a). There was a significant difference between the median number of oocysts between groups (Kruskal-Wallis one-way ANOVA, *H*_(4)_ =15.67, *P* = 0.0035, Fig. 1b). On day 17 PE, the mean number of sporozoites per mosquito ranged from 2490 in cohort C to 9730 in cohort A (Fig. 1c). There was a significant difference between groups as determined by one-way ANOVA (*F*_(4, 15)_ = 54.11, *P* < 0.0001). However, *post-hoc* analysis showed that there was no significant difference in sporozoite load between some groups. Consequently, for further analyses of the second experiment, the cohorts were grouped as high sporozoite load (AE), mid sporozoite load (B) and low sporozoite load (CD) where applicable.

### *Plasmodium falciparum* sporozoite RT-rtPCR detection threshold

To determine the RT-rtPCR assay’s limit of detection, a standard curve was prepared using RNA extracted from quantified sporozoites purified from mosquito salivary glands on day 17 PE. Serial dilutions of parasite RNA resulted in an *R*^2^ of 0.9451 and a slope of − 2.92, demonstrating the linear relationship between the logarithm of the number of parasites and C_t_ value within a 4-log_10_ dynamic range (Additional file 2: Figure S2). At a C_t_ value > 40 P. *falciparum* could not be detected.

### Mosquitoes excrete *P. falciparum* material soon after ingesting an infectious blood meal

**Fig. 2:**
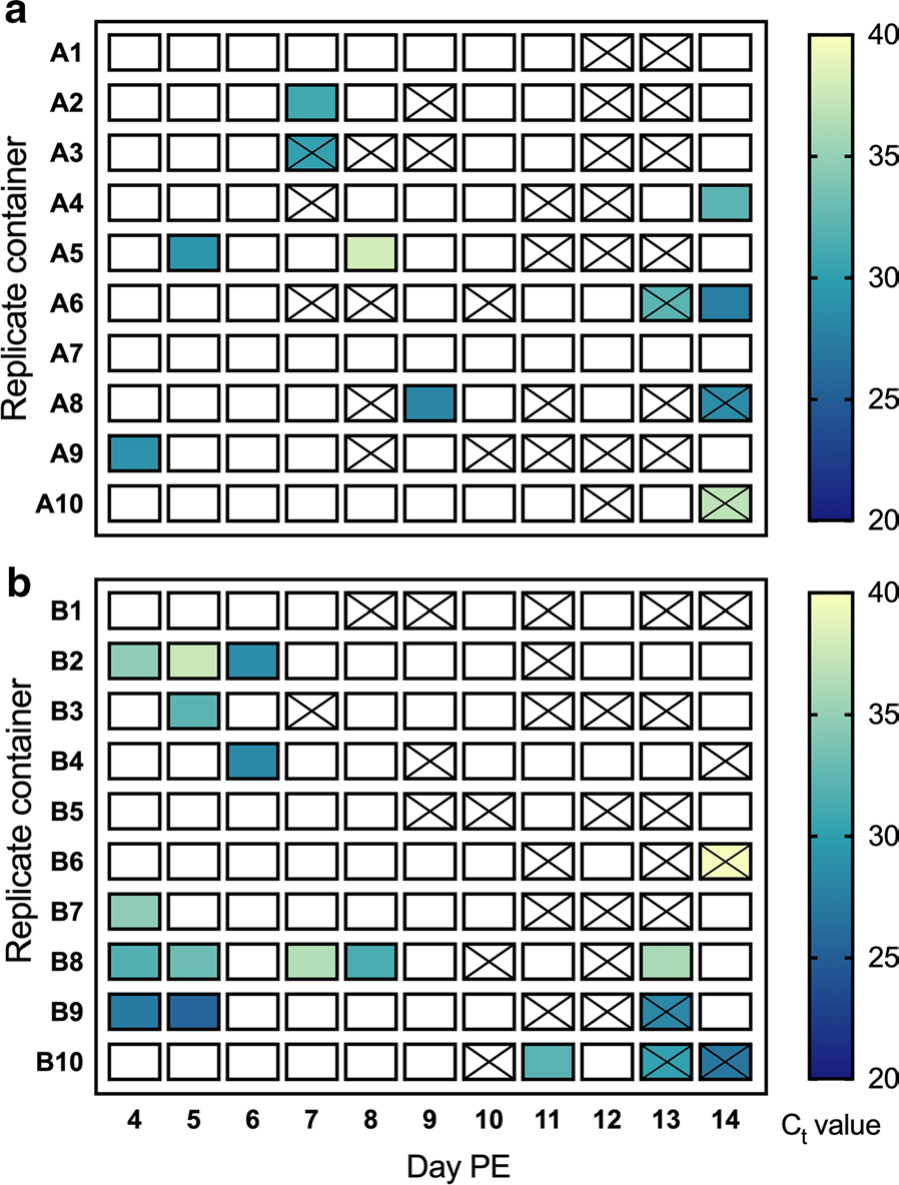
Time series RT-rtPCR detection of *P. falciparum* in excreta from groups of 5 *An. stephensi* mosquitoes. Excreta was collected daily from day 4 to 14 post-exposure (PE). **a** Mosquitoes with 68.2% oocyst rate and 9730 ± 910 sporozoites per mosquito. **b** Mosquitoes with 91.3% oocyst rate and 5630 ± 1460 sporozoites per mosquito. Lower C_t_ values correspond to a greater concentration of starting template; a blank cell indicates that *P. falciparum* RNA was not detected. An X indicates containers with no visible excreta spots

The excreta from 10 containers from each of 20 original containers from the two cohorts was analyzed over time. *Plasmodium falciparum* was detected in mosquito excreta by RT-rtPCR as early as day 4 PE in both cohorts (Fig. 2). For cohort A (68.2% oocyst rate and 9730 ± 910 sporozoites per mosquito), excreta samples collected from 8 out of 10 containers were positive at least once from day 4 to day 14 PE, with 10% (11/110) samples positive for *P. falciparum* overall. C_t_ values ranged from 27.5 to 37.9. For cohort B (91.3% oocyst rate and 5630 ± 1460 sporozoites per mosquito), excreta samples collected from 8 out of 10 containers were positive at least once for the duration of the experiment, with 16% (18/110) samples positive for the parasite and C_t_ values ranging from 25.5 to 39.7. No statistically significant difference was observed between mean C_t_ values between the cohorts (Two sample t-test, *t*_(2)_ = 0.5236, *P* = 0.6048). For both cohorts, no positive samples were observed on day 10 and day 12 PE.

### *Plasmodium falciparum* can be detected concurrently in mosquito excreta and saliva after sporogony

**Fig. 3:**
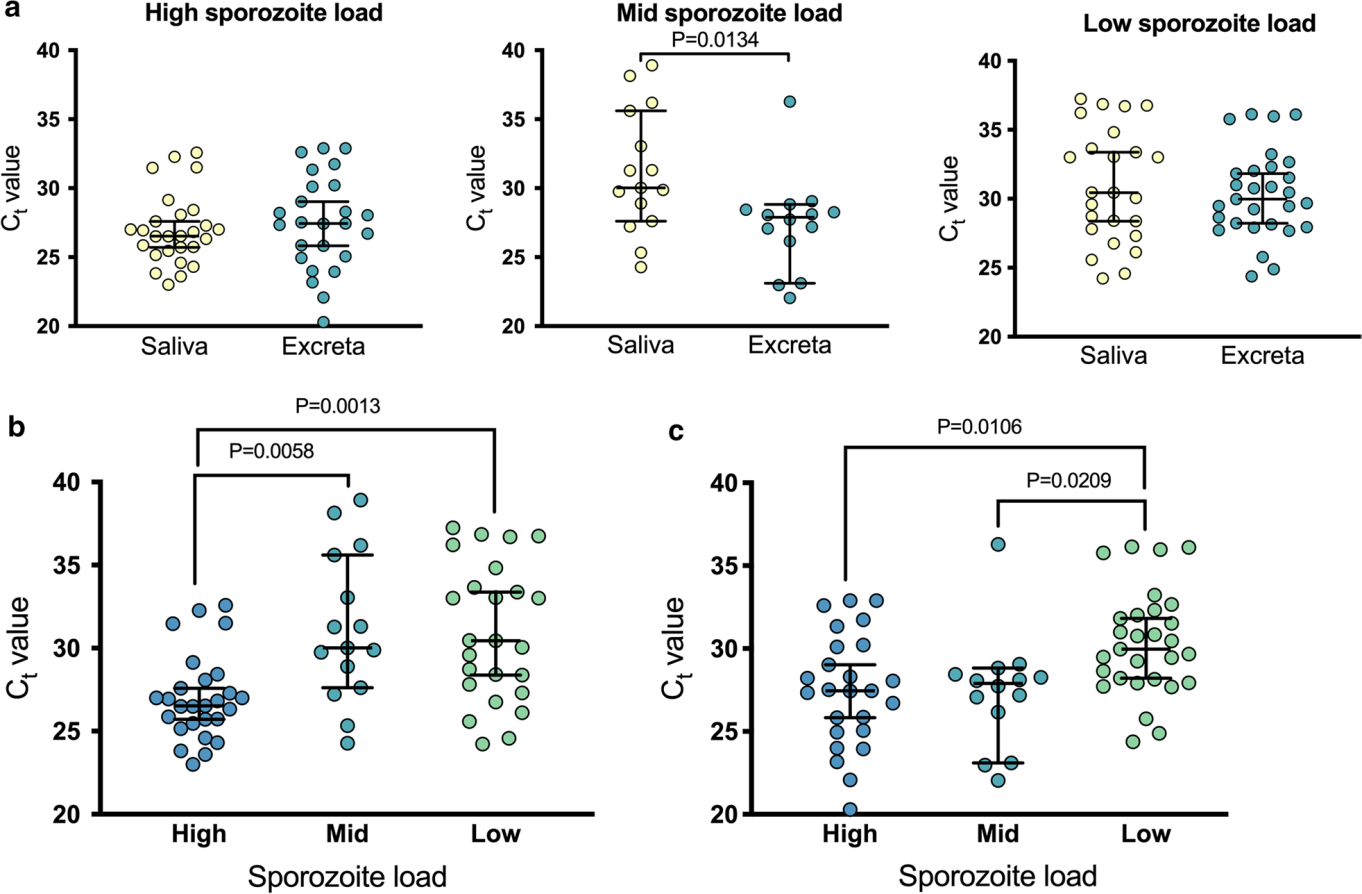
RT-rtPCR detection of *P. falciparum* in mosquito secretions collected from mosquitoes with high, middle and low sporozoite loads on day 15 to 19 post-exposure. **a** Detection of *P. falciparum* in saliva vs excreta in mosquitoes from the cohorts with different sporozoite loads. Wilcoxon matched-pairs sign ranked test. Detection of *P. falciparum* in saliva (**b**) excreta (**c**) from mosquitoes with different sporozoite loads. Kruskal–Wallis one-way ANOVA with Dunn’s multiple comparison test. Data are the median C_t_ value ± 95% CI. Each dot represents a group of 5 mosquitoes in a container. Lower C_t_ values correspond to a greater concentration of starting template

*Plasmodium falciparum* sporozoites were microscopically observed in the salivary glands of at least one mosquito removed from each of the containers analyzed in the second experiment (75/75). *P. falciparum* was detected by RT-rtPCR in 89% (67/75) of saliva samples and 91% (68/75) of excreta samples collected from day 15 to 19 PE, with no significant difference between these proportions (Fisher’s exact test, *P* > 0.9999). No significant differences were observed in median C_t_ values of saliva samples between days within the same cohort (Kruskal–Wallis one-way ANOVA, *P* > 0.05) or from excreta samples between days within the same cohort (Kruskal-Wallis one-way ANOVA, *P* > 0.05). Thus, the samples from different days from the same cohort were analyzed together from this point onward. No statistically significant differences were observed between median C_t_ values obtained from saliva and excreta, except the mosquitoes with a medium sporozoite load, where the median C_t_ value was lower in excreta than saliva (Fig. 3a; 27.9 *vs* 30.0, Wilcoxon matched-pairs signed rank test *W*_(14)_ = 14, *P* = 0.0134). When comparing detection between mosquitoes with different sporozoite loads, a statistically significant difference was observed in median C_t_ values from saliva (Fig. 3b; Kruskal–Wallis one-way ANOVA, *H*_(2)_ = 15.61, *P* = 0.0004). A statistically significant difference was also observed in median C_t_ values from excreta between these cohorts (Fig. 3c; Kruskal–Wallis one-way ANOVA, *H*_(2)_ = 11.39, *P* = 0.0034).

**Fig. 4:**
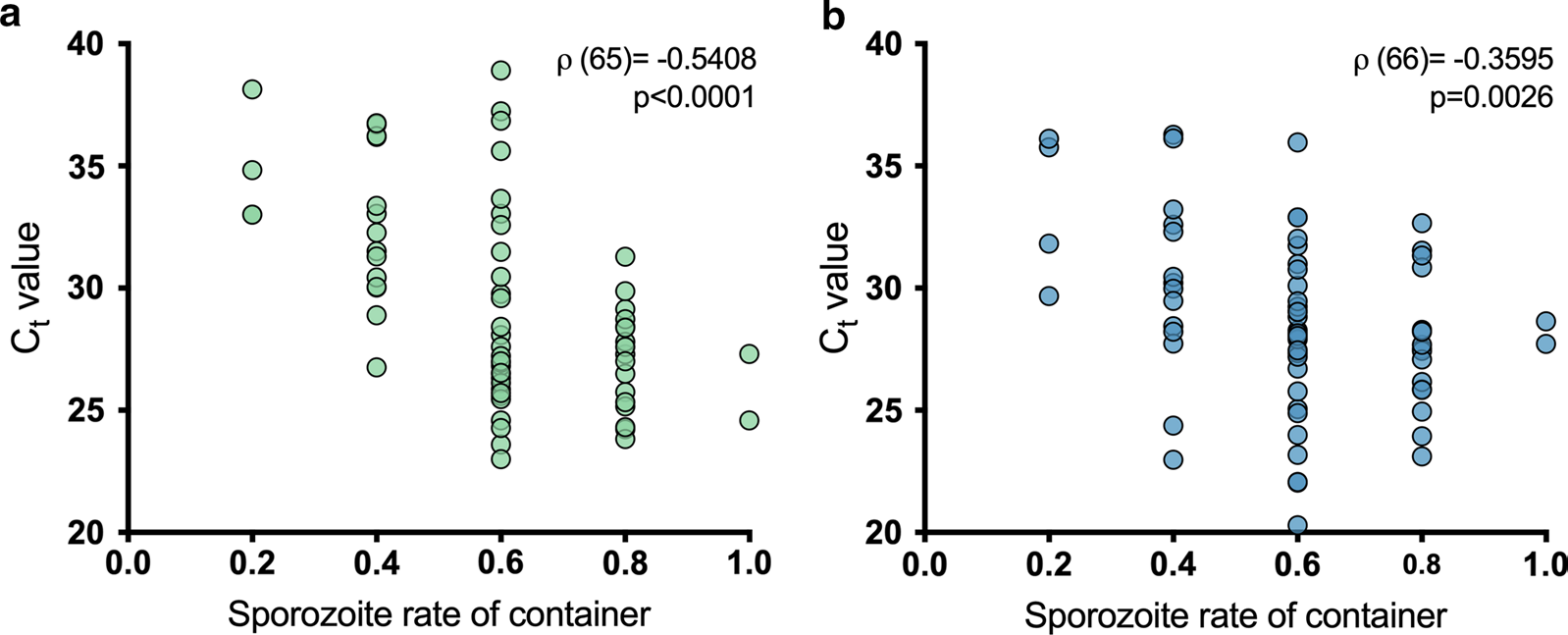
Spearman’s rank correlation between presence of sporozoites in salivary glands and RT-rtPCR detection of *P. falciparum*. Each dot represents a group of 5 mosquitoes in a container, sampled from day 15 to 19 post-exposure. Correlation between sporozoites and C_t_ values obtained from saliva (**a**) and excreta samples (**b**)

All containers from which saliva and excreta were harvested from had at least one mosquito with sporozoites in their salivary glands. The overall sporozoite rate for these mosquitoes was 60%, and the sporozoite rates were 66%, 56% and 54% for high, mid and low sporozoite load cohorts, respectively. A negative association was observed between the sporozoite rate of the container and the C_t_ value in saliva (Fig. 4a; Spearman’s rank correlation *ρ*_(65)_ = − 0.5408, *P* < 0.0001): the higher the proportion of mosquitoes with sporozoites in their salivary glands, the lower the C_t_ value (indicating higher amounts of the template). For excreta, this association was lower but still negative (Fig. 4b; Spearman’s rank correlation *ρ*_(66)_ = − 0.3595, *P* = 0.0026).

### Visualization of *P. falciparum* life stages in excreta samples

A subsample (10/68) of the excreta samples that were positive for *P. falciparum* by RT-rtPCR were examined microscopically in sextuplicate. No visual evidence of sporozoites or other life stage of the parasite was found in these samples.

## Discussion

Given the limitations of traditional methods to study mosquito-borne diseases, there has been concern in finding innovative or alternative samples for analysis. Mosquito saliva expectorated during sugar feeding has been used for research and surveillance of arboviruses [22, 23], and recently mosquito excreta has been proposed as a sample to enhance the sensitivity of saliva detection or for molecular xenomonitoring [25–28]. To our knowledge, this is the first study to evaluate the excretion and expectoration of *P. falciparum* in parallel with parasite development in the mosquito. Our results confirm previous findings that *Plasmodium* can be detected in mosquito excreta [28] and saliva deposited on filter paper cards after sugar feeding [24].

Previous studies have demonstrated that *P. falciparum* DNA can be detected in mosquito excreta on days 2–3 PE [29]. In this study, excreta was not collected until day 4 PE, to allow for blood meal digestion, which takes approximately 72 hours to be completed [33]. Our results indicate that *P. falciparum* nucleic acid in mosquito excreta continues to be detectable after blood meal digestion from day 4 to at least 19 PE. The source of the nucleic acid or the parasite life stage in excreta is unknown, but several hypotheses could explain its presence. Once a mosquito feeds on an infected host, it ingests gametocytes, the sexual stage of the parasite. An hour later, fertilization occurs, and by 24 hours the ookinete enters the midgut were oocysts establish and begin mitosis [34]. It has been suggested that some of the material excreted in the early days could be metabolized merozoites [28], the asexual parasites in the intraerythrocytic cycle, which are present in ingested blood meal in a ratio of about 156 merozoites per gametocyte [35] and which cannot infect, or survive in, the mosquito.

Additionally, early stage parasite development from ookinete to oocyst is closely related with blood-meal digestion; ookinetes that fail to traverse the midgut and transform to oocysts after digestion are destroyed [36]. From day 11 to 16, the oocysts burst producing thousands of sporozoites that migrate through the hemocoel to the salivary glands [37]. This is an inefficient process: some of these oocysts may be unsuccessful in producing sporozoites and the released sporozoites can fail to navigate, invade or survive in the salivary glands, with less than 20% of the sporozoites released by oocysts reaching salivary glands [38]. The remaining sporozoites are degraded in the hemocoel [39], and although the mechanism is unknown, it is possible that the residue finds its way to the Malpighian tubules to be excreted with other unwanted substances of the hemolymph. Although 40% and 91% of the containers sampled from day 11 to 14 and 15 to 19, respectively, were positive for *P. falciparum* by RT-rtPCR, no distinguishable parasites were observed under microscopy following Giemsa staining in any of the analyzed samples. Further studies of the contents of mosquito excreta are required to determine the source of the excreted nucleic acid.

We were able to detect *P. falciparum* sporozoites deposited on filter paper cards after sugar feeding on days 15 to 19 PE. Our results expand the results from Brugman et al., who detected sporozoites on cotton wool pledgets from day 18 to 24 [24]. It is likely that the differences in C_t_ values in saliva samples between cohorts are due to differences in sporozoite rates and not in sporozoite loads. The high sporozoite load cohort also had the highest sporozoite rate (66%) compared to the other cohorts (56% and 54% for mid and low sporozoite cohorts respectively). This was further demonstrated by the negative association between the C_t_ values obtained from saliva samples and the sporozoite rate of the container. Studies have suggested that the sporozoite load in the salivary glands is not a predictor for sporozoite transmission [40] because the structure of the salivary glands limits the number of sporozoites that are expectorated [21].

With the exception of the mid sporozoite load cohort, no significant differences were observed between detection of *P. falciparum* in excreta and saliva in samples collected after sporogony. It is interesting to note that although mosquitoes from this cohort had the highest oocyst rate (91%) at day 8 PE, the sporozoite rate from day 15 to 19 was moderate (56%) in the context of this experiment. This could explain the lower C_t_ values observed in excreta, since many of the sporozoites produced by the oocysts may have failed to reach the salivary glands, and may have been destroyed and possibly voided in excreta.

In this study we did not directly evaluate the detection of the parasite in secretions from individual mosquitoes. However, we were able to detect *P. falciparum* in the saliva and excreta from 80% of containers where just one mosquito had a salivary gland infection, indicating that the method is sensitive enough to detect the parasites from an individual mosquito. Previous studies have demonstrated that trace amounts of *Brugia malayi* DNA are detectable in samples that contain excreta from as many as 500 uninfected mosquitoes [28]. Similarily, it does not appear that the saliva of numerous uninfected mosquitoes affects the detection of arboviruses in mosquito expectorate. It is unlikely that saliva or excreta from many mosquitoes would interfere with the detection of *P. falciparum* from a single infected mosquito. However this needs to be further evaluated.

Detection *of Plasmodium* in mosquito excreta and saliva has applications in the laboratory. Observation of oocysts in mosquito midguts can be used as an estimation of mosquito infectivity [41]; however, oocysts are not visible until 6–7 days after ingesting an infectious bloodmeal, making it impossible to determine the infectious status of mosquitoes for a week [36]. Our results suggest that excreta could be monitored after bloodmeal digestion as soon as day 4, allowing for an earlier estimation of the potential of the parasite to establish a midgut infection in a non-destructive manner. An important component of vectorial capacity is the estimation of the period of sporogony, the period from which a mosquito ingests gametocytes to when it can transmit sporozoites to a receptive host [42]. Traditionally, this has relied on the detection of sporozoites in mosquito salivary glands. Monitoring the expectoration of the parasite could be a useful tool for exploring genetic traits and different environmental conditions that influence this period, allowing for a precise measurement of time-to-event in individual mosquitoes [43]. Genetic analyses and drug and vaccine development studies often rely in infected mosquitoes feeding on animal models, such as mice [44, 45] and non-human primates [46, 47]. Since our method allows for non-destructive screening of the parasite in the vector, mosquitoes that are transmitting could be pre-selected to increases the chances of transmission and potentially reducing the number of animals used in an experiment.

The analysis of mosquito saliva and excreta could also be implemented for malaria surveillance in the field. Currently, parasite detection in mosquitoes requires testing thousands of mosquitoes, either individually by microscopy or in pools by ELISA or molecular methods. Indeed, as transmission of a pathogen decreases, larger numbers of mosquitoes are necessary to improve the likelihood of capturing the less frequent occurrence of infection. Honey-based surveillance using nucleic acid preservation cards or wicks to collect mosquito saliva has been successfully incorporated by public health agencies in Australia and USA for routine surveillance of arboviruses [23, 48, 49] with several advantages over traditional methods. First, it reduces the number of samples that need to be processed down to 1–2 samples per trap. Secondly, the cards or wicks do not require a cold chain, making the method a logistically attractive approach. Finally, detection of the pathogen in mosquito saliva gives a better estimate of transmission risk, since only the mosquitoes that are transmitting will yield a positive result. Recently, it has been demonstrated that detection of arboviruses in excreta can be used to enhance the sensitivity of honey-based surveillance since the volume of the sample is larger [25, 26]. In the context of malaria surveillance, honey-based methods could be incorporated in regions with known co-circulation of malaria and arboviruses with the advantage of detecting all the circulating pathogens from one sample. Since it is not possible to determine how many mosquitoes expectorated or excreted in a trap, it is not possible to calculate an entomological metric, such as the sporozoite rate. Additionally, although a positive excreta result would not be sufficient to suggest that the mosquitoes in the trap are transmitting *Plasmodium*, it would indicate that the parasite is circulating. However, together with geolocation and mapping of larval habitats and areas of human activity [50], a positive result can be used to identify potential foci of transmission. This is particularly interesting in low transmission settings or to monitor re-establishment after elimination. In this study we used RT-rtPCR for pathogen detection, but the use of portable and automated rapid diagnostic test (RDT) devices for detection of the parasite in mosquito saliva and excreta samples needs to be assessed. Although the majority of RDTs available for *Plasmodium* focus on diagnosis of human samples [51], a VecTest™ dipstick assay for detection of sporozoites from mosquitoes has been developed [13, 14]. Dipstick assays have the advantage of providing results within minutes and do not require specialized equipment or infrastructure. Currently, a centrifugal microfluidic multiplex vector-diagnostic platform (LabDisk) to be used with mosquitoes is being evaluated [52]. The sensitivity of these assays is not as good as PCR-based detection, but given that collection of mosquito saliva and excreta is relatively simple, it could be coupled with RDTs or portable devices for use in low-resource settings and remote locations.

## Conclusions

The development of methods to estimate malaria transmission in low-transmission settings has been identified as one of the objectives by the malaria Eradication Research Agenda (malERA) [53]. As elimination targets are met, it is evident that novel approaches will be needed to ensure that transmission foci are identified, and re-establishment is prevented. Mosquito saliva and excreta have potential to be added to the array of samples supporting the crusade for malaria elimination and eradication. The samples are relatively easy to collect and can be used by surveillance programmes to detect evidence of malaria transmission, especially in low resource settings since the number of samples that need to be tested is reduced. Finally, as evidenced by studies of other mosquito-borne diseases, it appears that excretion of pathogens by infected mosquitoes is a general phenomenon that can be exploited for research and surveillance applications.

## Additional files

**Additional file 1: Figure S1.** Kaplan–Meier survival curves for cohorts of mosquitoes exposed to five different gametocyte cultures. The survival distribution was not different between cohorts (Log-Rank statistic $\chi^{ 2}_{\left( 3\right)}$ = 4.415, *P* = 0.220).

**Additional file 2: Figure S2.** RT-rtPCR standard curve. The standard curve was prepared using a suspension of *P. falciparum* sporozoites isolated from mosquito salivary glands. X-axis corresponds to the concentration of triplicate serially diluted template; Y-axis corresponds to RT-rtPCR C_t_ values.

## Data Availability

All data generated or analyzed during this study are included in this published article and its additional files.

## Abbreviations

ACT: artemisinin-based combination therapy
ELISA: enzyme-linked immunosorbent assay
PCR: polymerase chain reaction
PE: post-exposure
FP: filter paper
RT-rtPCR: real-time reverse transcription polymerase chain reaction
RDT: rapid diagnostic test
malERA: malaria Eradication Research Agenda
