# Supplementary figures and images for: Malaria surveillance from both ends: concurrent detection of Plasmodium falciparum in saliva and excreta harvested from Anopheles mosquitoes
Source: Parasit Vectors. 2019 Jul 18;12:355. doi: 10.1186/s13071-019-3610-9 (PMC6639908; doi:10.1186/s13071-019-3610-9)

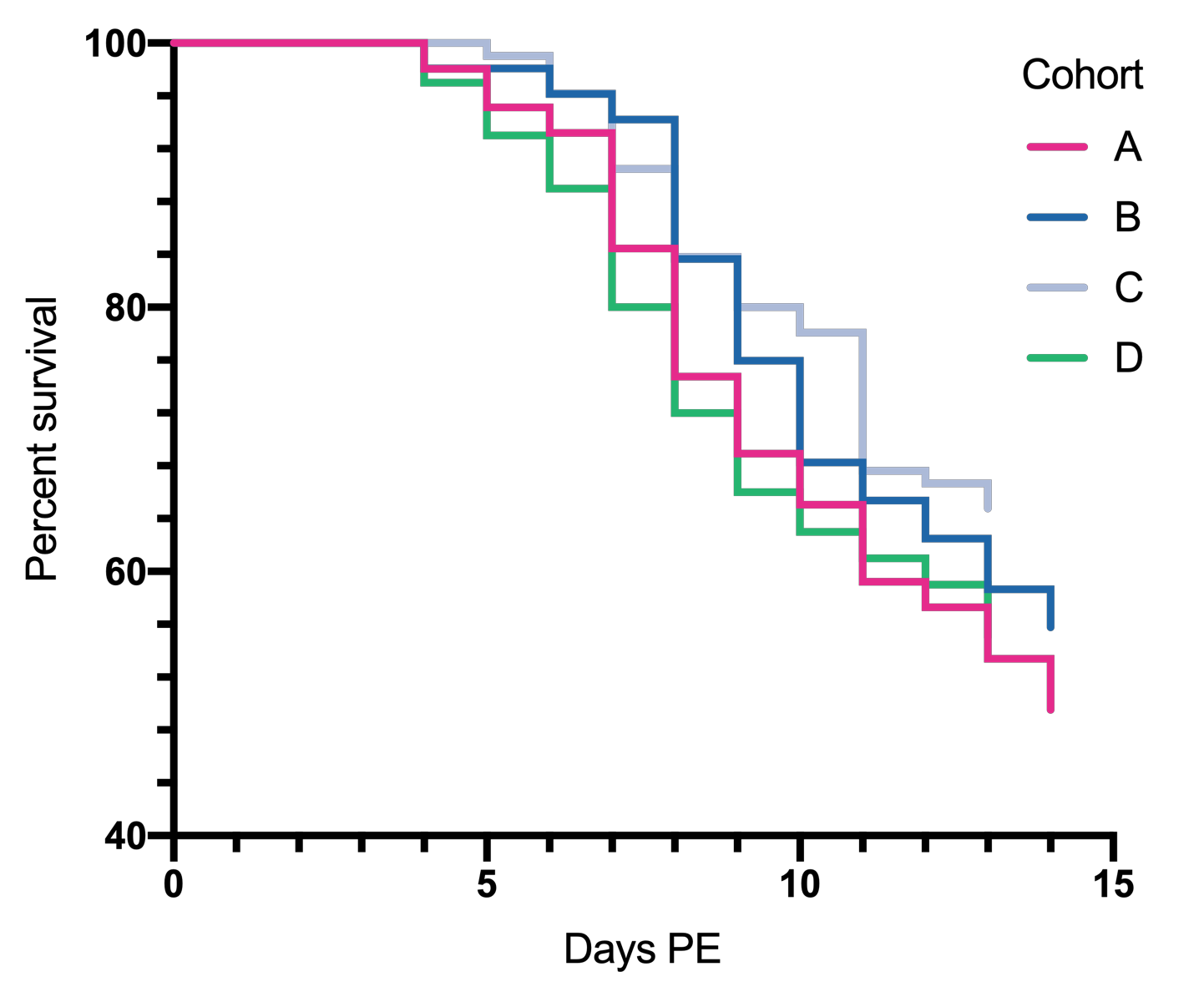

Supplement: Supplementary file 1 — Additional file 1: Figure S1. Kaplan–Meier survival curves for cohorts of mosquitoes exposed to five different gametocyte cultures. The survival distribution was not different between cohorts (Log-Rank statistic $\chi^{ 2}_{\left( 3\right)}$χ32 = 4.415, P = 0.220). [file 13071_2019_3610_MOESM1_ESM.tiff]

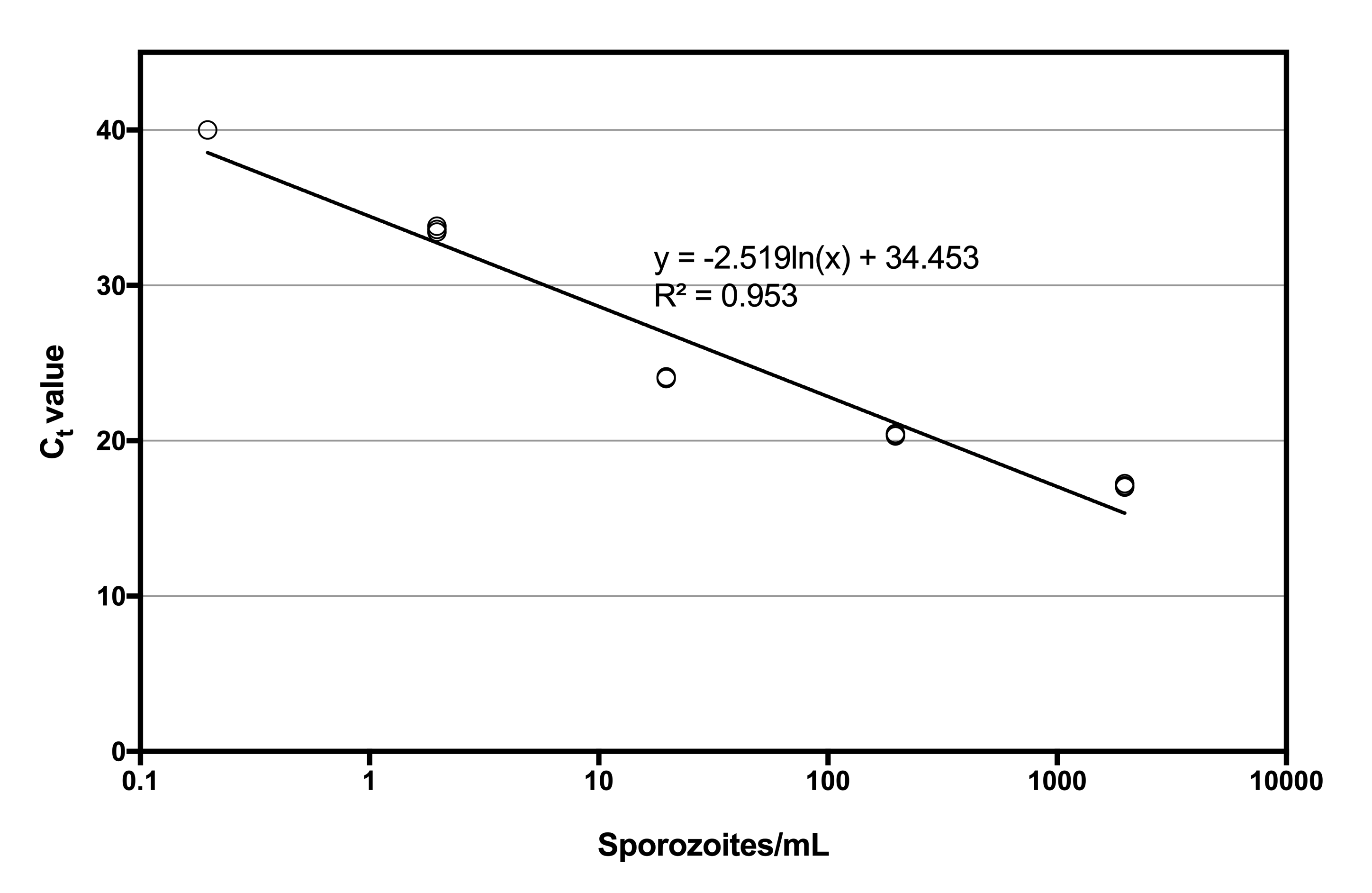

Supplement: Supplementary file 2 — Additional file 2: Figure S2. RT-rtPCR standard curve. The standard curve was prepared using a suspension of P. falciparum sporozoites isolated from mosquito salivary glands. X-axis corresponds to the concentration of triplicate serially diluted template; Y-axis corresponds to RT-rtPCR Ct values. [file 13071_2019_3610_MOESM2_ESM.tiff]
